# Is Five Percent Too Small? Analysis of the Overlaps between Disorder, Coiled Coil and Collagen Predictions in Complete Proteomes

**DOI:** 10.3390/proteomes2010072

**Published:** 2014-02-07

**Authors:** Zoltán Gáspári

**Affiliations:** Faculty of Information Technology and Bionics, Pázmány Péter Catholic University, Práter st. 50/A, H-1083 Budapest, Hungary; E-Mail: gaspari.zoltan@itk.ppke.hu; Tel.: +36-1-886-4780; Fax: +36-1-886-724

**Keywords:** intrinsically disordered protein, coiled coil, collagen triple helix, cross-prediction, proteome size

## Abstract

Identification of intrinsic disorder in proteins and proteomes has revealed important novel aspects of protein function and interactions. However, it has been pointed out that several oligomeric fibrillar protein motifs such as coiled coils and collagen triple helical segments can also identified as intrinsically disordered. This feature has not yet been investigated in more detail at the proteome level. The present work aims at the identification and quantification of such overlaps in full proteomes to assess their significance in large-scale studies of protein disorder. It was found that the percentage of cross-predicted residues is around 5% in the human proteome and is generally near that value in other metazoan ones but shows remarkable variation in different organisms. In particular, smaller proteomes are increasingly prone to such cross-predictions, thus, especially the analysis of viral proteomes requires the use of specific prediction tools.

## 1. Introduction

Intrinsically disordered proteins (IDPs) are widely considered to constitute a significant fraction of proteomes, especially eukaryotic ones [1,2]. Thus, the exact identification of IDPs and intrinsically disordered segments is an important aspect in large-scale structure-function studies. It has recently been pointed out that some structural elements, referred to as oligomeric fibrillar motifs below, are often predicted to be disordered by current IDP prediction algorithms [3,4]. Although the occurrence of structured motifs within low-complexity segments and other regions regularly predicted as IDPs has been noted [5], these are rarely taken explicitly into account when evaluating IDP abundance at the proteome level. However, in contrast to “genuine” IDPs which can be characterized by a diverse conformational ensemble in their native state [6,7], coiled coils, collagen triple helices and charged single alpha-helices are structured elements under physiological conditions. They also often exhibit functional roles characteristic for their structure, thus, their classification as intrinsically disordered is expected to be less informative than their specific identification and thus can be misleading. It should be noted, however, that coiled coil segments are likely disordered in their monomeric state [8,9] and this can also be hypothesized for collagen segments as they need specific trigger (usually coiled coil) segments to form the characteristic triple-helical structure [10]. 

Coiled coils are superhelical motifs consisting of alpha-helices wrapped around each other and having a characteristic helix-helix interaction referred to as knobs-into-holes packing [11,12]. This packing is a distinctive feature allowing us to set coiled coils apart from helical bundles. There are many different types of coiled coils, they can be double-, triple- or multiple-stranded, parallel and antiparallel and of lengths ranging from around 20 residues to several hundreds. Most commonly, coiled coil sequences can be identified based on a characteristic heptad repeat structure, but both irregularities and other types of repeats can complicate their detection. Coiled coils are specific oligomerization motifs involved, among others, in forming the cytoskeleton, governing membrane fusion and controlling gene regulation [13]. We have previously conducted a detailed study on coiled coil and IDP prediction algorithms and found that identifying coiled coils as IDPs is a common scenario [3]. 

The collagen triple helix consists of three polypeptide chains adopting the polyproline II helical conformation. Such conformation is preferred by segments rich in proline and glycine, more specifically, the prototypical sequence is formed by (POG)_n_ repeats, where O denotes hydroxyproline. Collagen triple helices most commonly occur in the collagens of connectivity tissues, but can also be found in other proteins as short trimerization motifs [14].

In this study, the extent of cross-predictions between IDPs, coiled coils and collagen triple helices is investigated in full proteomes. The term “cross-prediction” refers to the occurrence of segments or residues that are recognized both as disordered and coiled coil-forming or disordered and collagen helix-forming by the respective prediction algorithms. The most important question addressed is the extent of predicted IDPs that are better characterized as oligomeric fibrillar motifs and whether such cross-predicted disordered proteins/segments affect the general picture of IDP distribution. Although these aspects were previously investigated for specific protein sets [3,15], they were not yet addressed at the proteome level where the distribution of different protein types might largely influence the extent of cross-prediction.

## 2. Experimental

Full proteome sets investigated were compiled using the 2013_10 release of UniProt. All sequences with the keyword “Reference proteome” were extracted from the SwissProt and TrEMBL sections and were added to the protein set of the respective organisms. A further restriction for a reference proteome to be processed was that at least one of its constituent proteins must be annotated in SwissProt, leading to a total of 519 proteomes. Although all of these proteomes were processed, 211 viral protemes consisting of less than 10 proteins are typically not discussed below unless indicated otherwise (Table 1). The resulting set of proteomes is expected to be reasonably reliable and suitable for comparative analysis. 

**Table 1:** Summary of proteomes with at least 10 proteins used in this study.

Taxonomic group	Number of proteomes
Archaea	18
Bacteria	69
Embryophyta	10
Eukaryota (except Metazoa & Embryophyta)	49
Metazoa	57
Viruses	105
All organisms	308

To assess the potential bias in the results originating from redundancy, proteomes (except for the 211 viral ones with less than 10 residues) were filtered to the 40% level of similarity using the program PSI-CD-HIT (version 2012-0914) [16].

According to the suggestion formulated in our previous work [3], disordered segments were predicted with the program IUPred [17] using the long disorder option. Its output was processed so that only disordered segments of at least 30 residues were retained. Coiled coils were predicted using the program ncoils [18]. To account for the variability of prediction algorithms, predictions were also performed using VSL2B [19] and Paircoil2 [20] with similar criteria. Overlaps between IUPred and VSL2b as well as between ncoils and Paircoil2 were calculated using the segment overlap (SOV) measure [21] as described in [3]. SOV was calculated both for positively and negatively predicted segments (*i.e*., disordered/not disordered, coiled coil/not coiled coil) denoted SOV(+) and SOV(−) below. Consensus predictions of IUPred and VSL2B were generated using a minimum consensus IDP segment length of 30, and between ncoils and Paircoil2 using a minimum consensus coiled coil segment length of 21. 

Collagen segments were recognized with hmmer [22] using the HMM profile downloaded from the Pfam database [23].

Predictions were processed by generating masked FASTA files where each residue was replaced by a character indicating whether it was recognized by one or more prediction algorithms. These masked FASTA files were then used to extract various statistics about the extent of individual and cross-predictions. All processing steps were performed with in-house Perl scripts. 

## 3. Results and Discussion

Full results of the analysis are available as Supplementary material. When comparing prediction with and without cross-predictions, the terms “all disordered residues” and “pure disordered residues” will be used below. The former refers to all residues that are predicted to be disordered, whereas the latter only to those residues which are not also simultaneously predicted to be either in coiled coil or collagen regions. As can be expected based on the characteristic amino acid distribution of the latter two motifs, *i.e*., that prolines and glycines are underrepresented in α-helices but abundant in collagen triple helical motifs, no cross-predictions between coiled coils and collagen segments were detected in this study. 

### 3.1. Comparison of Predictions on Full Proteomes

**Figure 1:**
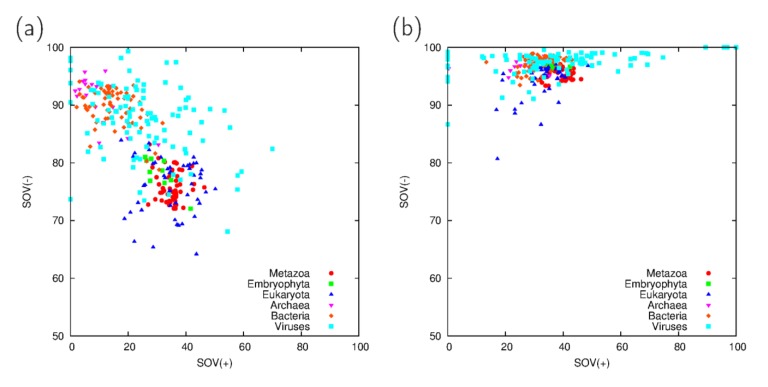
(**a**) Segment overlap (SOV) statistics of IUPred and VSL2B as well as (**b**) ncoils and Paircoil2 on the proteomes investigated. SOV(+) refers to the overlap between positive predictions (*i.e*., segments predicted to be in disordered or coiled coil regions by both respective prediction algorithms) whereas SOV(−) is the overlap between segments not recognized as disordered or forming coiled coils.

Disorder and coiled coil predictions were compared using the segment overlap (SOV) measure for both positively and negatively predicted segments in the proteomes investigated (Figure 1). As these prediction algorithms have different theoretical underpinnings, their overlap is rather limited for positive predictions (e.g., for IUPred and VSL2B this means segments predicted to be disordered). The previously noted high specificity and low number of predicted segments characteristic of coiled coil predictors [3] is in agreement with the high SOV(−) values observed for ncoils and Paircoil2.

Apparently, the overlaps between predictions vary considerably on the proteomes investigated, and the results remain qualitatively similar even for the 40% similarity filtered proteomes (data not shown). This suggests that the prediction algorithms are not universally applicable and their results depend on the exact composition of the proteome investigated. On the other hand, the high variation seen in viral proteomes is most likely attributable to the relatively low number of the proteins they contain and thus their diversity approximates that of individual sequences. 

Especially noticeable is the low agreement between the predictions obtained for bacterial and archaeal proteomes, whereas it is higher for all eukaryotic proteomes investigated. It should be noted that this could be, at least, due to the generally higher fraction of disordered residues in eukaryotes.

### 3.2. Fraction Residues Predicted to Lie in Disordered, Coiled Coil and Collagen Segments

**Figure 2:**
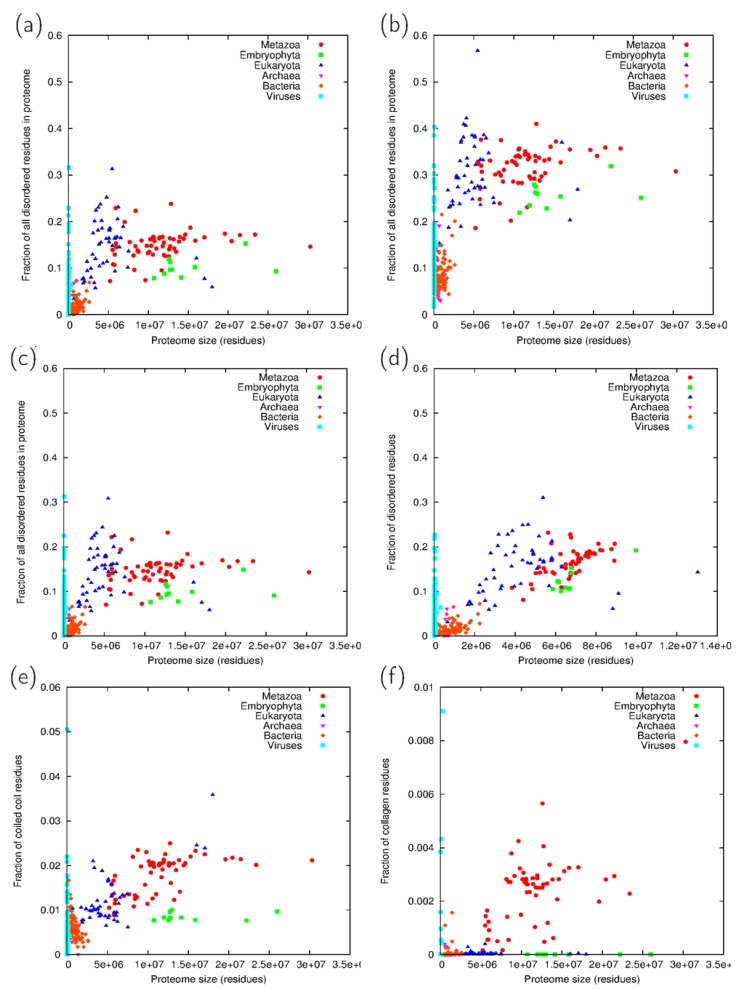
(**a**) Fraction of all residues predicted to be disordered by IUPred and (**b**) VSL2B and (**c**) their consensus; (**d**) Fraction of all residues predicted to be disordered according to the consensus prediction in the 40% filtered proteomes; (**e**) Fraction of all residues predicted to form coiled coils using the consensus of ncoils and Paircoil2; (**f**) Fraction of all residues predicted to be in collagen segments Data are depicted as a function of the size of the proteomes. Taxonomic groups are exclusive, *i.e*., Eukaryota means all eukaryotes except Metazoa and Embryophyta.

The individual outputs of the programs can be depicted as the fraction of residues predicted to be in the respective structural state as a function of proteome size (Figure 2). It is apparent that all three features are most abundantly represented in eukaryotes, but not necessarily metazoans. The most striking difference is between the number of disordered residues predicted by IUPred and VSL2B (Figure 2a,b), as VSL2B predicts approximately twice as many disordered residues than IUPred. However, the relative abundance of disorder is essentially the same between groups for both of the predictors and their consensus (Figure 2c). Embryophyta usually have a lower fraction of both disordered and coiled coil residues than metazoans (Figure 2c,d). Not surprisingly, only Metazoa show a measurable fraction of collagen segments (Figure 2e). However, it should be noted that the small viral genomes exhibit a high variability in each case, most likely reflecting the requirements imposed by their host organism. For example, the Sputnik virophage, a virus infecting *Acanthamoeba* cells already hosting a giant mimivirus [24] displays a percentage of collagen-predicted residues of 3.18; thus, it lies outside the plots shown in Figure 2f. 

### 3.3. Analysis of Cross-Predicted Residues

To address whether all residues predicted to be in coiled coils or in collagen segments were also predicted to be disordered, overlaps between the predictions were analyzed. As collagen segments can practically only be found in Metazoa, cross-predictions for this structure are also almost exclusively found in this taxon. For this group, the fraction of IDP-predicted residues in collagen-like segments does not exceed 6% in full proteomes and falls below 2% in filtered protein sets. However, the variability of disordered residues that are better characterized as coiled coils is much larger and characteristic of all taxonomic groups investigated. Interestingly, this fraction is characteristically around 4–5 percent for metazoans including the human, *Gorilla*, *Pan*, rat and mouse proteomes.

The highest extent of cross-predictions is found in some viral genomes, as much as 65% of IDP residues is predicted to lie in coiled coils in *Gammalipothrixvirus*, 56% in Bovine ephemeral fever virus, 34% in Cassava vein mosaic virus and 33% in *Salterprovirus*. Not surprisingly, the Sputnik virophage displays the highest percentage (42%) of disordered residues that are also predicted to be in collagen-like segments.

**Figure 3:**
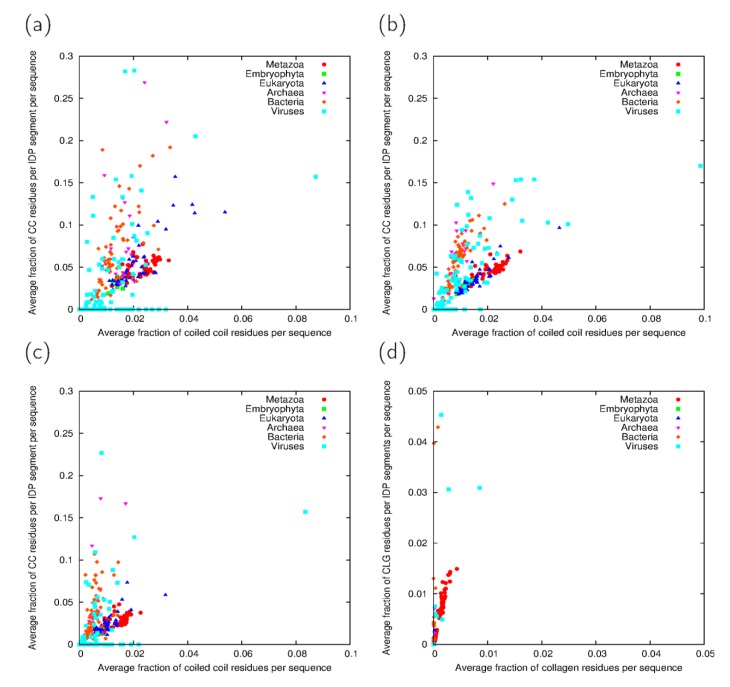
Comparison of coiled coil and collagen predictions for all sequences and within disordered segments. (**a**) Average number of coiled coil-predicted residues within IDP segments per sequence as a function of the average number of coiled coil residues per sequence using the IUPred-ncoils, (**b**) the VSL2B-Paircoil2 predictor pairs and (**c**) their consensus; (**d**) Average number of collagen-predicted residues within IDP segments per sequence as a function of the average number of collagen residues per sequence obtained with consensus prediction. Taxonomic groups are exclusive, *i.e*., Eukaryota means all eukaryotes except Metazoa and Embryophyta.

The relationship between all coiled coil residues predicted within a proteome and the extent of coiled-coil disorder cross-predictions varies by taxonomic group (Figure 3). In general, the extent of cross-predictions is higher than the average abundance of coiled coils in the full proteome, but the ratio of these is highest in bacterial proteomes in general and is closer to one in eukaryotes (Figure 3a–c). Again, viral proteomes exhibit the highest diversity. For collagen segments, the extent of cross-predictions is higher than the average abundance of such segments in Metazoa (Figure 3d).

**Figure 4:**
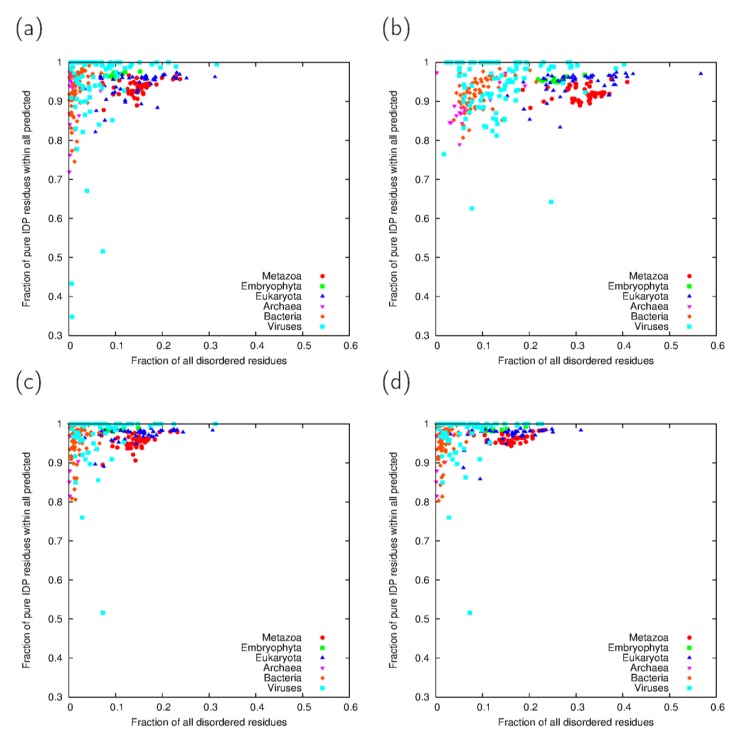
(**a**) Fraction of disordered residues not predicted to be in oligomeric fibrillar structures using the IUPred-ncoils, (**b**) the VSL2B-Paircoil2 predictor pairs and (**c**) their consensus. Fraction disordeed residues not predicted to be in oligomeric fibrillar structures in 40% filtered proteomes (**d**). Taxonomic groups are exclusive, *i.e*., Eukaryota means all eukaryotes except Metazoa and Embryophyta.

Considering all IDP-predicted residues, the fraction of them being in coiled coils and/or collagen segments varies up to 25% excluding viral proteomes (Figure 4). For metazoans, it is generally below 10% and typically around 5%–6%. The two eukaryotes with highest percentages are *Entamoeba histiolytica* and *Trichomonas vaginalis*, for both of which the cross-predictions are almost exclusively with coiled coil segments. As Embryophyta have practically no collagen-like sequences, the extent of cross-predictions in their case also comes from coiled coil segments, of which they have less than metazoans (Figure 2c,d). Interestingly, Bacteria show a high diversity in the extent of cross-predictions despite having a low percentage of general disorder in their proteome.

To get a more complete picture on the effect of cross-predictions on extent of predicted disorder in proteomes, segments of at least 30 residues long were considered with and without considering cross-predictions. It is apparent that although cross-predictions lower the number of such segments, the number of pure IDP segments correlates well with those without considering cross-predictions (Table 2). This observation means that the presence of coiled coil and collagen regions does not affect the general picture of disorder in large-scale comparative investigations on the relative abundance of disordered segments across proteomes.

In sharp contrast with this, the number of pure and all coiled coil segments shows predictor-dependent correlation in some proteomes, especially in Embryophyta where excluding cross-predictions with VSL2B causes a considerable discrepancy with all coiled coil predictions (Table 3). This observation again suggests that prediction outputs cannot be uniformly interpreted in all contexts and points to the need of using multiple predictions and consensus methods wherever possible.

**Table 2:** Correlation between the number of all and pure disordered segments (≥30 residues) in full and 40% filtered proteomes by predictor pair and taxonomic group.

Taxonomic group	IUPred-ncoils		IUPred-Paircoil2		VSL2B-ncoils		VSL2B-Paircoil2		Consensus	
	Full	40%	Full	40%	Full	40%	Full	40%	Full	40%
Archaea	1.000	1.000	1.000	1.000	0.998	0.998	0.999	0.999	1.000	1.000
Bacteria	1.000	1.000	1.000	1.000	1.000	0.999	1.000	1.000	1.000	1.000
Embryophyta	1.000	1.000	1.000	1.000	1.000	1.000	1.000	1.000	1.000	1.000
Eukaryota	0.997	0.998	0.999	0.999	0.999	0.999	0.999	0.999	0.999	0.999
Metazoa	0.999	0.999	1.000	0.999	1.000	0.999	1.000	1.000	1.000	0.999
Viruses	0.999	0.999	0.999	0,999	0.999	0.999	0.999	0.999	0.999	0.999

**Table 3:** Correlation between the number of all and pure coiled coil segments (≥21 residues) in full and 40% filtered proteomes by predictor pair and taxonomic group.

Taxonomic group	IUPred-ncoils		IUPred-Paircoil2		VSL2B-ncoils		VSL2B-Paircoil2		Consensus	
	Full	40%	Full	40%	Full	40%	Full	40%	Full	40%
Archaea	0.917	0.910	0.907	0.912	0.546	0.518	0.652	0.642	0.894	0.896
Bacteria	0.926	0.923	0.976	0.977	0.646	0.693	0.842	0.857	0.917	0.914
Embryophyta	0.991	0.946	0.995	0.932	0.879	0.063	0.911	0.053	0.990	0.916
Eukaryota	0.985	0.976	0.995	0.992	0.957	0.926	0.981	0.965	0.991	0.986
Metazoa	0.987	0.949	0.995	0.973	0.941	0.763	0.977	0.885	0.989	0.963

### 3.4. Relevance of Cross-Predictions

As no prediction algorithms are perfect, it can be expected that there are some erroneous predictions for each proteome. The question arises whether the observed cross-predictions are simply a result of such errors. We have previously conducted a thorough analysis on the performance of disorder and coiled coil prediction algorithms with focus on cross-predictions. The accuracy of the disorder predictions was considerably more variable than that of coiled coil predictions, which is line with the more regular—thus more easily recognizable—sequential features of coiled coils. Cross-predictions preferentially occurred in the form of coiled coils being recognized as disordered segments but not the reverse. Thus, in agreement with other considerations in the literature [25], it can be proposed that the more specific coiled coil and collagen predictions should have precedence over “general” disorder predictions. In this context, the finding that the overlap between fibrillar motifs and disorder predictions varies for organisms and even taxonomic groups strongly suggests that these do not entirely come from random errors but reflect actual variations in proteome composition.

## 4. Conclusions

Our results show that although the disorder content of most proteomes can be reliably estimated by using disorder predictions only, more detailed studies should take into account the possible presence of oligomeric fibrillar motifs, especially in metazoans, although in these organisms the extent of cross-predictions can be safely assumed to be around 5%. However, special care should be taken for viral proteomes, bacteria and for some non-metazoan eukaryotes as in these the extent of cross-predictions can be relatively high, reaching as much as 15%–20% or even more in some viruses.

We note that there might be other oligomeric fibrillar motifs that might have to be considered, for example, the charged single alpha-helix (CSAH) [26], which is estimated to be present in about 0.2% of proteins in the human proteome [27]. Thus, although CSAHs can be cross-predicted both as coiled coils and as disordered sequences [3,26], their presence is not likely to influence the outcome of proteome-level studies of disorder. 

The results presented here are in line with the fact that individual protein sequences can display high variation in the extent of cross-predictions, thus, smaller proteomes such as bacterial and, more prominently, viral ones are also non-uniformly affected. Thus, while the general picture of the distribution of intrinsic disorder can be reliably assessed for most eukaryotic proteomes without considering oligomeric fibrillar motifs, the analysis of small proteomes, like that of individual proteins, requires the simultaneous use of more specific prediction tools. 

## Supplementary Materials

Supplementary File 1
